# Supplementary material for: Insights into hydrothermal treatment of biomass blends: Assessing energy yield and ash content for biofuel enhancement
Source: PLoS One. 2024 May 22;19(5):e0304054. doi: 10.1371/journal.pone.0304054 (PMC11111091; doi:10.1371/journal.pone.0304054)
Supplement: Attachment — Submitted filename: Rebutal letter 13042024.docx [file pone.0304054.s001.docx]

Dear Academic Editor and Reviewers,

This document details the changes made to address the observations and suggestions provided for the manuscript. We deeply appreciate the time you have dedicated to reviewing the document.

Best regards,

Dr. Fidel Vallejo

**Academic Editor**

1. **Please ensure that your manuscript meets PLOS ONE's style requirements, including those for file naming. The PLOS ONE style templates can be found at
   https://journals.plos.org/plosone/s/file?id=wjVg/PLOSOne_formatting_sample_main_body.pdf and 
   https://journals.plos.org/plosone/s/file?id=ba62/PLOSOne_formatting_sample_title_authors_affiliations.pdf**

We have thoroughly reviewed the document and are pleased to confirm that it adheres to PLOS ONE's style requirements.

2. **Please expand the acronym "DICYT" and "UNACH" so that it states the name of your funders in full.
   This information should be included in your cover letter; we will change the online submission form on your behalf.**

The information was included in the cover letter.

3. **We note that your Data Availability Statement is currently as follows: All relevant data are within the manuscript and its Supporting Information files.**

**Please confirm at this time whether or not your submission contains all raw data required to replicate the results of your study. Authors must share the "minimal data set" for their submission. PLOS defines the minimal data set to consist of the data required to replicate all study findings reported in the article, as well as related metadata and methods (https://journals.plos.org/plosone/s/data-availability#loc-minimal-data-set-definition).**

**For example, authors should submit the following data:**

**- The values behind the means, standard deviations and other measures reported;
- The values used to build graphs;
- The points extracted from images for analysis.**

**Authors do not need to submit their entire data set if only a portion of the data was used in the reported study.**

**If your submission does not contain these data, please either upload them as Supporting Information files or deposit them to a stable, public repository and provide us with the relevant URLs, DOIs, or accession numbers. For a list of recommended repositories, please see https://journals.plos.org/plosone/s/recommended-repositories.**

**If there are ethical or legal restrictions on sharing a de-identified data set, please explain them in detail (e.g., data contain potentially sensitive information, data are owned by a third-party organization, etc.) and who has imposed them (e.g., an ethics committee). Please also provide contact information for a data access committee, ethics committee, or other institutional body to which data requests may be sent. If data are owned by a third party, please indicate how others may request data access.**

We confirm that our submission contains all the raw data necessary to replicate the results of our study, as per PLOS requirements. Also, the raw data is now accessible in the Kaggle repository: 10.34740/kaggle/dsv/8029353.

**Reviewer 1**

**Introduction**

**- The sub-heading of Residues revalorization and Related work should be removed from the introduction. The detail for these can be summarized under the head of introduction.**

The sub-headings of *Residues revalorization* and *Related work* were removed and included within the introduction.

**- Line 50: 4.4 ·1011 kWh should be revised as 4.4 x 1011 kWh**

The requested modification was made (line 92)

**- Line 50: ….imported energy, please check it is energy or fuels. Energy means electricity, …., heat.**

The text was revised, and the word 'energy' has been changed to 'fuels' as indicated in the suggestion.

**- Line 51: check the unit of 22.4 million m3. Is it suitable for biomass?**

The request was reviewed, and the measurement unit for biomass is appropriate. Biomass can be expressed in mass or volume units, such as cubic meters, due to its ease of measurement and relevance to efficient waste and resource management. Although the standard unit of measurement for energy calculations is the kg or ton, the waste projection is based on the space it could occupy (in dumps and landfills), which implies knowing a volume. In other studies, the unit was in volume: (Pryshliak & Tokarchuk, 2020, p. 24), "animal manure and poultry manure in Ukraine amount to 900 million m^3^ of cattle manure," and (Yamamoto et al., 2019, p. 337) "Annual supply potential of timber and forest biomass resources was 1,070,234 m^3^".

The references are in the following DOIs: 10.2478/environ-2020-0003 and 10.1080/14942119.2018.1514178.

**- Line 60: the definition of waste should be provided before. For this case, waste covers the materials from municipal solid waste (MSW) (organic and inorganic components) and biomass (only organic materials)? This is because the incineration is normally applied for MSW. Management of MSW and biomass is different, the authors need to be clear for this issue.**

We reformulated the introduction section to clarify this issue.

**- Line 65: the function of gasification is not for producing heat, but for generating synthesis gas or syngas, which can be applied to produce heat, power and others.**

In the text, we specified one of the advantages of gasification, which produces heat by using the properties of the syngas generated. We modified the statement as follows (line 57):

*"Gasification generates syngas which presents certain advantages, such as the ability to produce heat simultaneously and serve as raw material for biorefineries."*

**- Line 67: tar formation is not because of biomass moisture, but it is because of another component and reaction. The authors need to be clear for this issue.**

We reformulated the phrase and introduced references to clarify this issue.

**- The clear definition of hydrothermal carbonization (HTC) and its advantages are required. It is only in subcritical water?**

Hydrothermal carbonization (HTC) exclusively occurs under subcritical water conditions. The advantages of HTC are mentioned in the text (introduction section), as follows:

"*The carbon densification achieved through this process can reach up to 50% compared to the raw biomass [11]. The final product, hydrochar, exhibits enhanced calorific values, hydrophobic properties [12], and greater ease of transport and storage [13]. On average, the improvement in calorific values can reach up to 30 to 40 % compared to untreated biomass [14]. The low reaction temperature (180–250 ̊ C) and high-moisture biomass conversion without a prior dewatering and drying process are its main advantages over other thermo-chemical conversion technologies [15]."*

**- The advantages and drawbacks of HTC should be provided, as well as the comparison between torrefaction.**

The advantages are mentioned in the text as we mentioned above.

The main drawbacks are mentioned in the text as follows:

"*However, the effects of different input process variables on* *hydrochar quality are still being studied, aiming for biomass to be effectively used to produce renewable energies [16]."*

Regarding torrefaction:

*"A similar situation arises with torrefaction, a process akin to pyrolysis, conducted under inert conditions and temperatures of up to 300°C"... "In contrast, hydrothermal carbonization (HTC) involves the treatment of materials in subcritical water, and a prior drying stage is unnecessary."*

**- The effects of some salts on insight characteristics of hydrochar should be summarized, particularly the slagging and fouling effects, and ash meting behavior.**

We introduce it in the introduction section, reinforcing the novelty of this research.

**- Effects of minor and major elements for blended biomass during HTC should be summarized.**

The suggestion has been taken into consideration.

The next quotation in the text acknowledges the importance of utilizing biomass blends to mitigate the variability in composition, thereby enhancing the efficiency of the HTC process:

"*High variability in biomass composition and its seasonal and geographical availability could be diminished using mixtures, which is crucial for this process [21].*"

**- The novelty of this work should be clearly indicated.**

Thank you for your remarks. The introduction was modified to show the novelty of this work clearly.

**Materials and methods
- Line 116: what is the definition of pure biomasses?**

Pure biomasses correspond to biomass samples originating from a specific source of biomass residues. We introduce it to clarify this issue in the Materials and Methods section.

**- The drawing or figure of HTC system is required, as well as its components.**

We did not include an image of the HTC system since the equipment was presented in a previous publication. The system and its components are available at <https://doi.org/10.1371/journal.pone.0291838.> This reference was included in the text.

**- The size of bulk density of biomass should be reported.**

Done. These values have been added in the Table 1.

**- The blended samples should be done based on dried mass. The water or moisture should be balance before the process. It should be same for all conditions.**

It is correct; the calculations related to the mass balance in the mixtures must be done on a dry basis, and this was pointed out in the Methodology section: *"Each charge biomass mixture (dry basis) adhered to the weight composition detailed in Table 2."*

**- How the authors determine the lignocellulosic components of biomass both before and after HTC.**

The lignocellulosic content was determined only for the raw materials. We added the methods applied for the lignocellulosic analysis as follows:

*"The lignin content was determined according to the TAPPI T 222 method. The cellulose and hemicellulose content were obtained using the Kushner-Hoffer and Jaime-Wise methods."*

After the HTC process, identification of the biomass macrocomponents is impossible due to the reactions occurring in the aqueous medium, mainly denaturation and depolymerization of these natural polymer chains.

**- The pressure of HTC at each temperature should be provided.**

Pressure is a consequential parameter. The text included this explanation to enhance clarity: " *The reactor operated under autogenous steam pressure which was generated during the reaction. While this parameter does not significantly affect the process itself, it's noteworthy that autogenic pressure increases with rising reaction temperature (Petrović et al., 2024)*".

**- The authors need to check this "proximate analysis was performed by the Van Soest method". Is it correct for proximate components?**

We modified the sentence as follows:

The lignin (L) was determined according to the TAPPI T 222 method. The cellulose (C) and hemicellulose (H) content were obtained using the Kushner-Hoffer and Jaime-Wise methods.

**- The unit of proximate and ultimate analyses should be clearly indicated as % wt. as received basis, dry basis, dry ash free basis….**

Thank you for your advice. Table 1 was modified accordingly.

**- The minor elements such as K, Cl, Mg, Si, Mn,…which related to slagging and fouling formation, as well as ash deformation should be determined and reported.**

The reviewer's suggestion is considered for future work since the evaluation of the ash components could provide clues to the kinetics of the HTC process. However, for the present work, and like similar studies, the ash is analyzed as a total since we try to know the influence of the operating conditions and biomass mixtures on its final content in the hydrochar, which is key for the scaling of this technology and the resolution of associated problems at a pilot level.

**- The number of digits should be two throughout the manuscript.**

Done.

**- Check the summation of each proximate result, and each ultimate result. Is it 100 % or not? If not, what happen?**

In all cases, the structural components (lignin, cellulose, hemicellulose), extractives (lipids, volatile and short chain compounds), and total ashes add up to 100%. However, for the ultimate results, the sum of Carbon, Hydrogen, Nitrogen, and Oxygen is not 100 % since it has been determined that agroindustrial organic waste contains significant quantities of inorganic elements such as K, Cl, Ca, Mg, and others, used in plant growth as nutrients.

(https://www.sciencedirect.com/science/article/abs/pii/S0016236120320056)

In summary, the total number of individual components (CHONS) does not add up to 100% because extensive ash characterization is needed, which is not within the scope of this study.

**Results and discussion
- More discussion related to the effects of operating parameters on thermal decomposition of lignocellulosic components is required.**

In the Results section, more recent literature has been incorporated, and the behavior of the variables has been explained more clearly in comparison with similar available studies.

**- The change in mass yield of the product is because of thermal decomposition, or dispersion of sample in hot water? The organic or volatile matter in water after HTC should be determined and reported, and discussed.**

It is common to determine the content of fixed carbon and volatile material before the HTC process. However, the process achieves close to 100% conversion of the carbon in the hydrochar, eliminating short-chain compounds and soluble matter, and therefore, the amount of C in the hydrochar increases concerning raw or untreated biomass. That is why the main focus of the analysis falls on the behavior of the structural compounds. This team demonstrated in previous works that they constitute the main factor in explaining the trends in the results.

**- The effects of minor elements in biomass samples on thermal decomposition during HTC are required to explain.**

They do not have any palpable effect since they are part of the ash that acts as an inert without reaction during the HTC process. While the fate of the ashes has been explored (https://www.sciencedirect.com/science/article/abs/pii/S0961953412005028, https://www.sciencedirect.com/science/article/abs/pii/S0016236115012594), studies from https://www.sciencedirect.com/science/article/abs/pii/S0016236120320056 indicate that in lignocellulosic biomass, Silicon and its salts are more than 40% of the total ash present and that you are according to https://www.sciencedirect.com/science/article/abs/pii/S0961953412005028 are removed at temperatures greater than 260 °C, which are not included in the range of the present study precisely because they cause an abrupt decrease in mass yield, caused by the decomposition reaction and short chain polymerization of lignin (Vallejo, 2020). On the other hand, the same study by Toufiq Reza 2013 and supported by the conclusions of Smith 2016, states that heavy metals leave the solid in the aqueous solution, which contains other elements and major compounds, such as furfurans and others. This ultimately has a very minor impact on the results analyzed. However, the reviewer's observation will be considered for future studies where ash kinetics can be addressed to eliminate possible problems in pilot or industrial-scale operations.

**- The remaining minor elements (K, Cl, Si, Mn, Mg,…) in biomass after HTC should be reported and discussed.**

The reviewer's suggestion is taken into consideration for future work since for the present work, and like similar studies, resources were limited for the specific analysis of minor elements, and the main objective was to understand the influence of the operating conditions and biomass mixtures on the final ash content in the hydrochar and its calorific value, which is key for the scaling of this technology and the resolution of associated problems at a pilot level.

**- The authors did not perform the optimization by RSM or another optimization technique, so the word "optimization" should be removed from the title.**

Thank you for your suggestion. We changed the title to "Insights into Hydrothermal Treatment of Biomass Blends: Assessing Energy Yield and Ash Content for Biofuel Enhancement" to avoid misunderstanding since the original title referred to the improvement of characteristics of a biofuel.

**- If the MY is reported as dried mass, is it important to use HHV for calculating EY?**

As stated by **equation 1**, the mass yield is calculated using the dry masses of the hydrochar and the unprocessed biomass. The Higher Heating Value (HHV) was determined based on dry hydrochar; thus, the increase in calorific power does not consider the moisture content of the solid in any case.

Finally, the Energy Yield (EY) is mathematically calculated from these laboratory-obtained values.

**Reviewer 2**

**1) Please justify why choose factorial design for design of experiment (DOE)? There are many DOE can be used to predict optimum conditions and to evaluate effects of parameters.**

The two-level factorial design is used to approach this study based on the following assumptions and limitations of the study:

1) The types of biomass mixture, the nature of the additive, and its dosage were categorical variables, so quadratic or central point designs cannot be applied.

2) This study aimed to analyze the broad region of experimentation in the sense of being able to affirm, if necessary, the validity of the model with some subsequent tests. However, the results indicated that the region could be described mathematically with the equations obtained from this design, with an R2 of 0.95 and 0.94 for groups 1 and 2, respectively. Compared with the literature, it is common to find increases in calorific value between 15 to 25%. In this work, the increase in calorific value was up to 40%, so the study was considered positive. It also focused on the reduction of the ashes, which has been shown to have an inverse behavior in the sense that an increase in severity in the process (temperature and time) generates a considerable increase in the calorific value but also increases the ashes, caused by the significant decrease in the mass yield at temperatures greater than 260 °C. For this reason, the analysis of the influence of the variables carried out in this study indicates that increases in times and temperatures generate a greater amount of ash.

Reviewing the literature, the study by a traditional DoE has yielded good results, https://www.mdpi.com/2071-1050/16/1/338, since the first-order interaction between the significant variables can explain the process.

**2) Please discuss on the error analysis.**

The Mean Absolute Percentage Error (MAPE) was presented in addition to the Anderson-Darling test. The findings indicate that the response equations in Tables 4 and 5 were appropriately adjusted to the experimental data.

**3) The authors are advised to discuss on the validation of the DOE. Additional experiments need to be conducted to validate the optimization results.**

The MAPE was presented in addition to the Anderson-Darling test. The findings indicate that the response equations in Table 4 and 5 were appropriately adjusted to the experimental data.

**4) Please compare your findings with literature.**

The results section has been modified to incorporate comparisons with findings available in the literature, which help explain the observed behavior of the variables.

**5) Characterization study? Need to discuss on the characterization study.**

In each table, a paragraph has been added analyzing the values shown and comparing them so that the reader of the article has greater clarity about the study's objective.
